# AOSLO-net: A Deep Learning-Based Method for Automatic Segmentation of Retinal Microaneurysms From Adaptive Optics Scanning Laser Ophthalmoscopy Images

**DOI:** 10.1167/tvst.11.8.7

**Published:** 2022-08-08

**Authors:** Qian Zhang, Konstantina Sampani, Mengjia Xu, Shengze Cai, Yixiang Deng, He Li, Jennifer K. Sun, George Em Karniadakis

**Affiliations:** 1 Division of Applied Mathematics, Brown University, Providence, RI, USA; 2 Beetham Eye Institute, Joslin Diabetes Center, Department of Medicine and Department of Ophthalmology, Harvard Medical School, Boston, MA, USA; 3 McGovern Institute for Brain Research, Massachusetts Institute of Technology, Cambridge, MA, USA; 4 School of Engineering, Brown University, Providence, RI, USA; 5 Division of Applied Mathematics and School of Engineering, Brown University, Providence, RI, USA

**Keywords:** adaptive optics scanning laser ophthalmoscopy images, deep neural networks, image segmentation, retinal microaneurysms, transfer learning

## Abstract

**Purpose:**

Accurate segmentation of microaneurysms (MAs) from adaptive optics scanning laser ophthalmoscopy (AOSLO) images is crucial for identifying MA morphologies and assessing the hemodynamics inside the MAs. Herein, we introduce AOSLO-net to perform automatic MA segmentation from AOSLO images of diabetic retinas.

**Method:**

AOSLO-net is composed of a deep neural network based on UNet with a pretrained EfficientNet as the encoder. We have designed customized preprocessing and postprocessing policies for AOSLO images, including generation of multichannel images, de-noising, contrast enhancement, ensemble and union of model predictions, to optimize the MA segmentation. AOSLO-net is trained and tested using 87 MAs imaged from 28 eyes of 20 subjects with varying severity of diabetic retinopathy (DR), which is the largest available AOSLO dataset for MA detection. To avoid the overfitting in the model training process, we augment the training data by flipping, rotating, scaling the original image to increase the diversity of data available for model training.

**Results:**

The validity of the model is demonstrated by the good agreement between the predictions of AOSLO-net and the MA masks generated by ophthalmologists and skillful trainees on 87 patient-specific MA images. Our results show that AOSLO-net outperforms the state-of-the-art segmentation model (nnUNet) both in accuracy (e.g., intersection over union and Dice scores), as well as computational cost.

**Conclusions:**

We demonstrate that AOSLO-net provides high-quality of MA segmentation from AOSLO images that enables correct MA morphological classification.

**Translational Relevance:**

As the first attempt to automatically segment retinal MAs from AOSLO images, AOSLO-net could facilitate the pathological study of DR and help ophthalmologists make disease prognoses.

## Introduction

Diabetic retinopathy (DR), a frequent complication of diabetes, remains the leading cause of new cases of blindness, among working-age adults in the United States,^1^ and it is expected to affect approximately 14.6 million people in United States by the year 2050.^2^ DR results in pathological alterations in both neural and microvascular structures and can damage any part of the central or peripheral retina.^3^^,^^4^ Based on the Early Treatment Diabetic Retinopathy Study severity scale, eyes with DR progress through a severity spectrum of DR from nonproliferative DR.^5^ Classification of these severity stages is performed by evaluating certain clinical features of the retina, such as microaneurysms (MAs), intraretinal hemorrhages, hard exudates, cotton wool spots, intraretinal microvascular abnormalities and venous beading.^6^ Advanced and vision-threatening stages of DR are characterized by neovascularization and by the presence of vessels that may leak fluid and eventually cause diabetic macular edema, which is manifested as thickening of the central retina.

**Figure 1.**
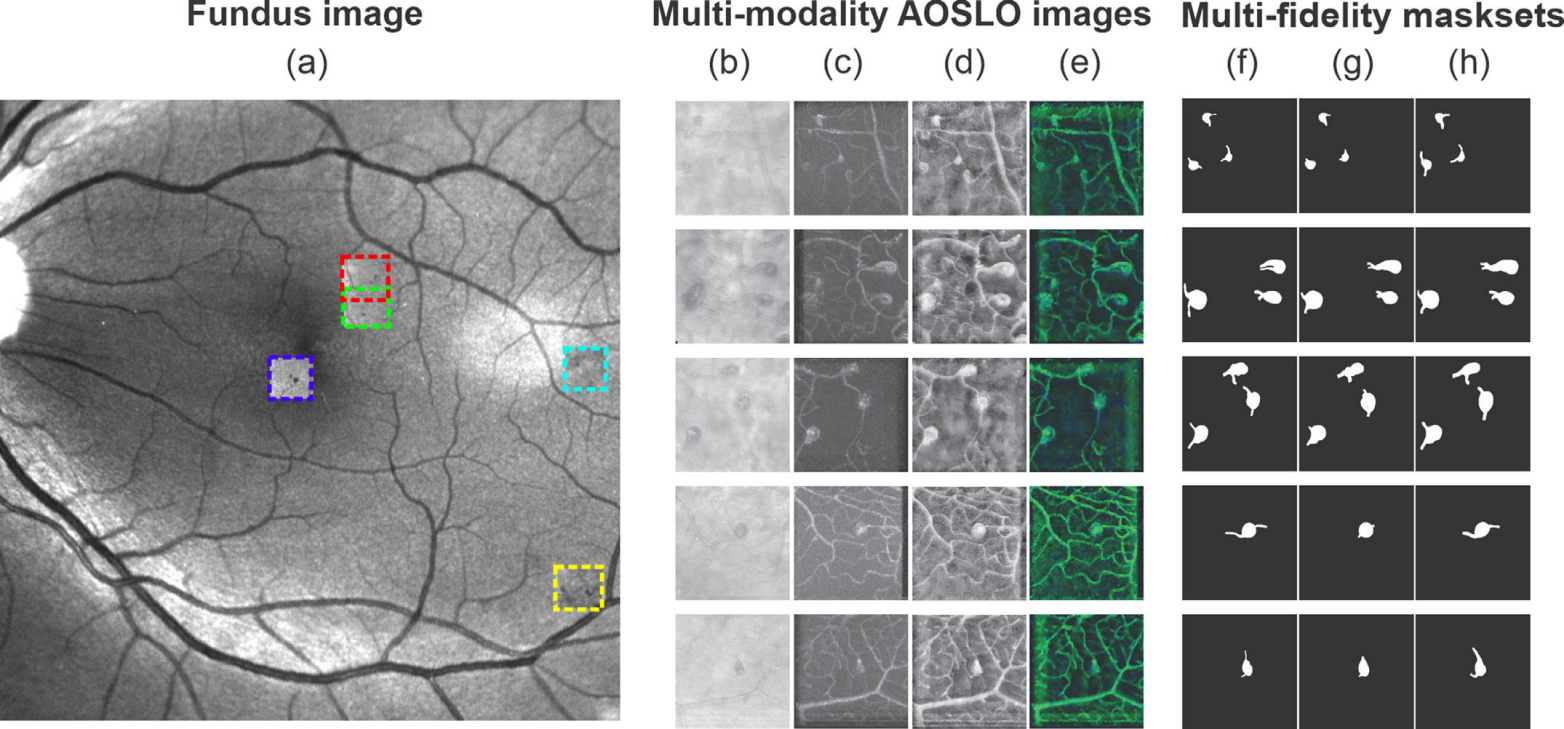
Multi-modality AOSLO images and multifidelity mask sets are used to train and test the AOSLO-net. (a) Five MAs imaged by AOSLO (highlighted by boxes in different colors) superimposed on a digital fundus photograph from an eye with diabetic retinopathy. (b–e) Examples of four sets of AOSLO images with different modalities used to train and test the AOSLO-net: (b) raw images extracted from the AOSLO videos; (c) perfusion maps (also see Supplementary Fig. S1); (d) preprocessed AOSLO images with enhancement; (e) two-modality images which are generated by concatenating the perfusion maps (c) and enhanced AOSLO images (d). Details of how multimodality AOSLO images are generated can be found in the Method section. These images illustrate that a varied number of MAs can be detected in a single AOSLO image. Images in row 1–3 contain multiple MAs, whereas images in row 4-5 contain a single MA with complicated background vessels whose size may be comparable to the MA. (f–h) Three sets of masks are generated independently to examine the robustness of the AOSLO-net to mask sets with different qualities. Normal set (f): masks are created based on both the AOSLO videos and perfusion maps to illustrate the body of MAs and their feeding and draining vessels. Short set (g): masks are designed to show shorter feeding and draining vessels of MAs compared to the normal set, while the thickness of the vessels remains similar to the normal masks. Thick set (h): masks are designed to show thicker feeding and draining vessels of MAs compared to the normal set, while the length of the vessels remains similar to the normal masks.

Fundus photography, a conventional and noninvasive imaging modality, has been widely used since the 1960s to access the severity of DR on the basis of the presence and severity of retinal vascular lesions.^7^^,^^8^ In fundus images (Fig. 1a), MAs appear as black circular dots with sharp margins whereas intraretinal hemorrhages, which also display as black dots or blots, may be larger and more irregularly shaped than MAs.^9^ MAs and intraretinal hemorrhages are frequently not able to be distinguished on fundus images as the standard fundus photographs do not provide microscopic details of these lesions or allow determination of vascular perfusion. Indeed, the Early Treatment Diabetic Retinopathy Study severity scale lumps the grading of these lesions into a single “H/MA” category. In contrast, more advanced imaging modalities, such as optical coherence tomography angiography^10^^–^^13^ and adaptive optics scanning laser ophthalmoscopy (AOSLO),^14^^,^^15^ is capable of providing higher-resolution details of these abnormalities and address the presence or absence of blood flow, thereby allowing differentiation hemorrhages from MAs.^16^ AOSLO provides retinal imaging with the highest resolution of all the available retinal imaging techniques on human retina (down to the cellular level [2 µm]) and thus has been used to identify and quantify the morphology of individual MA^14^ (Figs. 1b–e) and to measure blood flow at the capillary level.^17^^,^^18^

MAs are one of the earliest clinically visible signs of DR. MA leakage or rupture may precipitate local pathology in the surrounding neural retina that impacts visual function. Recent optical coherence tomography angiography and AOSLO-based studies have suggested a possible correlation between the morphology of retinal MAs and their tendency to leak fluid, rupture or form thrombus.^15^^,^^19^^,^^20^ These findings imply that accurate identification of the shapes of MAs might be useful in the future to improve DR prognosis. However, existing models for MA segmentation and classification have been trained on standard fundus photographs and therefore can only predict the number of MAs and their locations,^21^^,^^22^ because the resolution of standard fundus photography is not sufficient to analyze the shape of individual MAs. In contrast, the AOSLO imaging technique provides ultra-high resolution retinal images that can be used to classify MA morphologies.^14^ To date, AOSLO retinal images have been analyzed manually by specially trained personnel^14^^,^^15^^,^^19^^,^^23^ as no model has been developed to automatically process these images.

Emerging interest in automated analysis of retinal images has been sparked by the rapidly increasing prevalence of diabetes worldwide and the consequent need for scalable approaches to screen and triage patients at risk for vision loss from DR.^24^^–^^26^ With the recent advances in the computational power of graphics processing units, deep convolutional neural networks (DCNNs) have become a widely used tool for efficient DR screening.^27^ DCNNs are more generic compared to conventional methods that rely on hand-crafted features because the deep layers in the network act as a set of increased levels of feature extractors that can learn directly from the input images.^28^^,^^29^ Moreover, DCNN models are highly discriminative in automated DR severity grading and thus have achieved higher screening accuracy than conventional methods.^24^ In particular, UNet, combining an encoder and a decoder to form a “U-shape” structure, is a specially designed convolutional neural networks (CNN) architecture for biomedical image segmentation tasks. UNet is very effective in few-shot prediction with only a few labeled images when combined with data augmentation,^30^ and it has outperformed the plain DCNN in segmenting biomedical images, particularly for those with complex subtleties.^30^ Recent development of UNet has given rise to a number of variants, such as deformable UNet,^31^^,^^32^ residual UNet,^33^ recurrent residual and UNet,^33^ which further improved the segmentation accuracy on fundus images.

**Figure 2.**
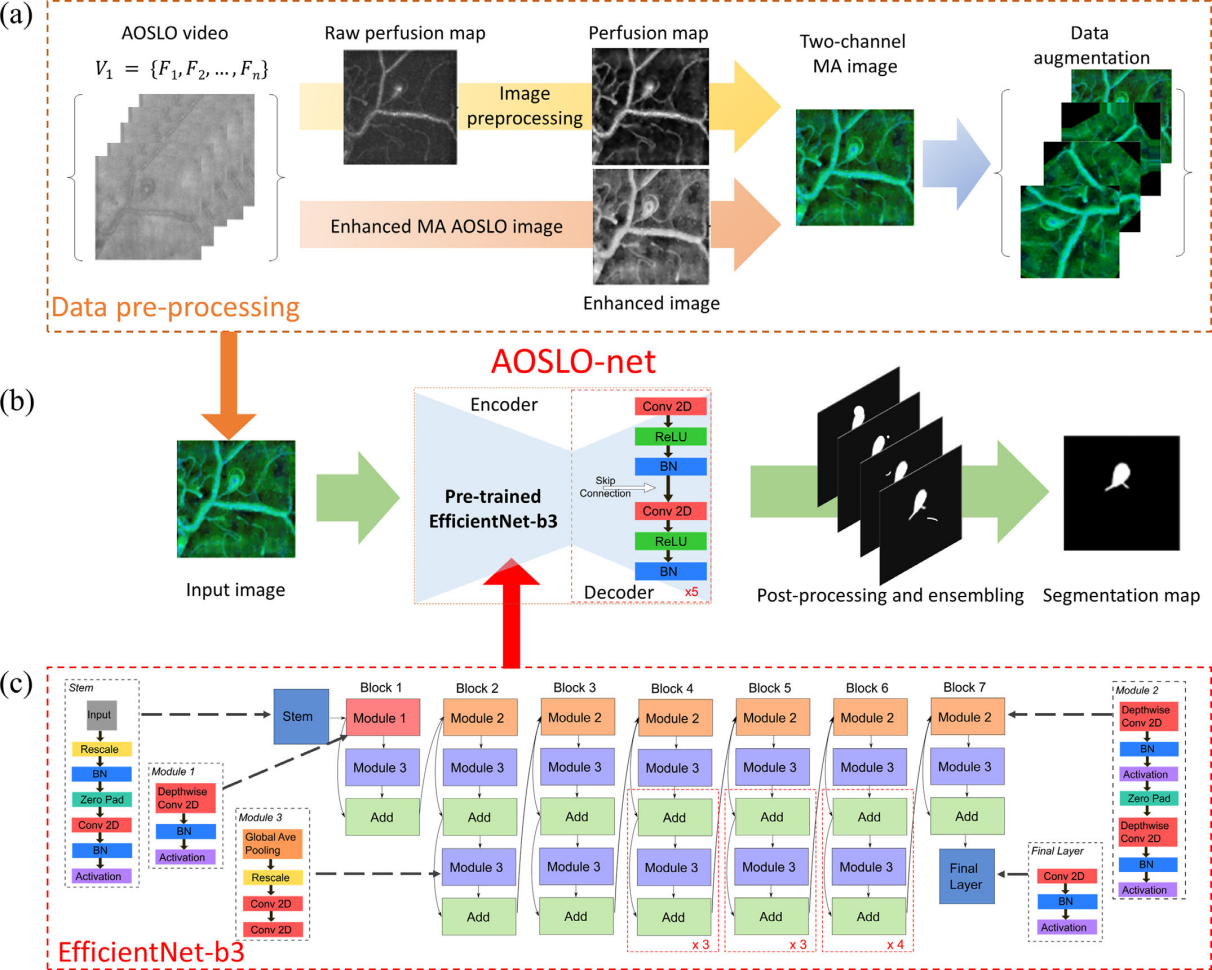
Overview of the architecture of AOSLO-net. The MA segmentation is performed through the following steps: data preprocessing, deep neural network training and inference, postprocessing and output ensembling. (a) Pre-processing AOSLO images for training the AOSLO-net. We first created the perfusion map by computing the deviations from the AOSLO video. Moreover, we created another set of enhanced AOSLO images from the AOSLO video. These two sets of images are concatenated to generate a third set of two-channel MA images. Data augmentation was performed on the two-channel MA images to increase the diversity of the training set (see examples in Supplementary Fig. S2). (b) Detailed modular components of the AOSLO-net (i.e., preprocessed multimodality images) produced from (a) are fed into the AOSLO-net, which consists of an EfficientNet-b3 encoder (c) and a regular UNet decoder. We then perform postprocessing and ensembling on the output images of the AOSLO-net to generate the segmentation map. (c) Detailed architecture of the EfficientNet-b3, which works as the “encoder” in the proposed AOSLO-net.

We have noted several features from the AOSLO image dataset that pose challenges for the existing automatic segmentation models. As shown in Figure 1b, (i) the contrast between the MA body and background is low whereas the level of the background noise is high; (ii) the boundary of the MA is not clearly defined; (iii) a typical AOSLO image may contain one or multiple MAs with different shapes and sizes; and (iv) there are numerous background blood vessels in the images, some of which are even at similar size as the MAs. These background vessels may interfere with the segmentation of the feeding or draining vessels of the MAs, which are crucial to determine MA morphology. In this work, we design the first deep neural network model, or AOSLO-net (Fig. 2), to perform automatic segmentation of MAs from AOSLO images and quantify their shape metrics that can be used for classification of MAs into different types, such as focal bulging, saccular, fusiform, mixed saccular/fusiform, pedunculated, and irregular-shaped MAs.^14^ The objective of this work is fundamentally different from some recent studies, which either use the AOSLO technique to segment microvessels without MAs^34^^,^^35^ or focus on segmenting MAs from fundus images,^22^^,^^36^^,^^37^ which do not provide the resolution required to identify the shape of MAs. This model is trained and tested by using 87 AOSLO MA images with masks generated manually by ophthalmologists or trained graders, the largest published AOSLO image dataset for this kind of effort thus far. We evaluated the performance of this model by comparing the model predictions with nnUNet,^38^ a state-of-the-art UNet model whose superiority has been demonstrated for dozens of publicly available databases.

## Methods and Materials

### Data Set

In this study, 87 MAs were imaged from 28 eyes of 20 subjects with varying severity of DR (56% nonproliferative DR and 44% PDR). Sixteen (80%) subjects had type 1 diabetes, seven (35%) of the subjects are female, and average age is 41*.*9 ± 10*.*4 years old, with mean diabetes duration 23.9 ± 8.4 years, and mean HbA1c is 8*.*2% ± 1*.*1%. Informed written consent was obtained from each participant before the performance of any study procedures at a single visit in Beetham Eye Institute, a tertiary referral center for diabetes care. This study adhered to the tenets of the Declaration of Helsinki and was approved by the institutional review board of the Joslin Diabetes Center.

Eighty-seven MAs from the eyes of adult study participants with diabetes underwent AOSLO imaging. All MAs were located within ∼20° of the foveal center. The AOSLO system has been previously described in detail by Lu et al.^39^ This system uses confocal and multiply scattered light imaging modes, and achieves a field size of ∼1*.*75° × ∼1*.*75° with lateral resolution of ∼2.5 µm on the retina. Moreover, 75-frame videos of each MA were aligned and averaged (MATLAB; The MathWorks, Inc., Natick, MA, USA). The magnification factor on AOSLO images was determined by eye axial length measurement or derived from the spherical equivalent of the eye. For this exploratory study, we included MAs with high quality AOSLO images (Fig. 1b), where the two-dimensional multiply scattered light images and corresponding perfusion maps (Fig. 1c) provided sufficient detail to identify the full extent of MAs’ bodies and their parent vessels’ boundaries.

The masks in our dataset, as illustrated in Figures 1f–h, are considered as the ground truth for training the AOSLO-net, and they are created manually by ophthalmologists and skilled trainees using ImageJ.^40^ Both the AOSLO images (Fig. 1b) and the corresponding perfusion map (Fig. 1c) are referred when masks are generated. Moreover, different groups of MA masks, as shown in Figures 1g and 1h, are created by varying the length and thickness of the parent vessels to test the robustness of AOSLO-net to masks with different qualities. The normal mask set (see Fig. 1f), which is generated by ophthalmologists, intends to represent the true geometries of the parenting vessels illustrated on the AOSLO images. The short mask set (Fig. 1g) is designed to show shorter feeding and draining vessels of MAs compared to the normal mask set, whereas the thickness of the vessels remains similar to the normal masks. The thick mask set (Fig. 1h) is designed to show thicker feeding and draining vessels of MAs compared to the normal set, whereas the length of the vessels remains similar to the normal masks. This examination also demonstrates the human ability of segmentation when comparing one kind of mask with another, because they equivalently represent the MAs.

### Image Preprocessing

As shown in Figure 1b, the raw AOSLO images are featured with intense background noise and low contrast. Thus we perform imaging preprocessing on these raw images and generate multimodality images to improve the effectiveness of the training. First, we generate a set of perfusion maps by tracing the blood flow in the MAs and micro-vessels using the pixel-by-pixel standard deviation method on different frames of the AOSLO video.^15^ As shown in Figure 1c, the vessels with blood motion appear bright in the perfusion map, while static tissue shows up as dark background. To improve the quality of the perfusion maps, we apply the following methods to denoise and enhance the images: (1) we use fast nonlocal means method to remove the background noise; (2) use normalization to make the pixel value lie in the interval (0*,* 1); (3) apply contrast limits adaptive histogram equalization (CLAHE) to enhance image contrast without over stretching the contrast in specific areas and balance the overall contrast; (4) apply Gamma Correction to remove some bright stripes on the background surrounding tissues caused by CLAHE. The impact of each of these four preprocessing steps on the raw perfusion maps is illustrated in Supplementary Figure S1.

Perfusion maps may not be able to accurately illustrate the geometries for all MAs because thrombosis may occur in some MAs, causing presence of nonperfused areas.^15^^,^^20^ Therefore we have developed enhanced AOSLO images to provide more details on the boundaries of each MA, as shown in Figure 1d. The procedure of creating enhanced AOSLO images follows three main steps: (i) taking the average over all the frames in MA video; (ii) reversing image color; (iii) performing local mean filtering. We note that in some enhanced AOSLO images, the boundaries of MAs and their parenting vessels are not clearly illustrated due to the low quality of the AOSLO images. Therefore we further generate a two-channel image set by concatenating the perfusion map and the enhanced AOSLO images (Fig. 1e), which use the information of the blood flow inside MAs to compensate for the missing information of the MA boundaries.

Because of the limited size of the AOSLO data set, we use data augmentation to increase the number of images for training the AOSLO-net. We apply three types of transformations, including flip, rotate and scaling, to the AOSLO images (perfusion map, enhance AOSLO and two-channel images) and their corresponding masks. The augmentation procedure follows three steps: (1) Images are flipped horizontally and vertically with probability of 0.5. (2) Flipped images are rotated with angles in the set

$$\begin{array}{c} \left\{0,\;\frac{2\pi }{N},\;\frac{4\pi }{N},\;\cdots ,\;\frac{2\left(N-1\right)\pi }{N}\right\} \end{array}$$

where *N* is selected to be 32. (3) The rotated images are scaled with a factor randomly selected between 0.7 and 1.4 to improve the robustness of AOSLO-net on segmenting MAs with varying sizes. Typical examples of augmented images are shown in Supplementary Figure S2.

### Architecture of AOSLO-net and Network Training

Inspired by the popular UNet structure,^30^ AOSLO-net is composed of two key parts: encoder and decoder. The function of the encoder is to extract the features of MAs at different levels whereas the decoder integrates these extracted features to compose the segmentation results. Because the role of the encoder is critical to the performance of the segmentation model, we adopt the current state-of-the-art image classification network EfficientNet-b3^41^ with a depth of 5 as the encoder in the AOSLO-net. On the other hand, the decoder in AOSLO-net contains 5 blocks, each of which contains two convolution layers with kernel size 3. The channel number of these blocks are (256*,* 128*,* 64*,* 32*,* 16). We also apply transfer learning in AOSLO-net through pretraining the EfficientNet-b3 using ImageNet^42^ to achieve quick convergence during training. The evolution of the loss in the training process can be found in Supplementary Figures S4, S5 and S7.

The preprocessed AOSLO images are split into five folds. Although one fold is reserved as test data, the remaining four folds are used to train and validate the AOSLO-net. Considering that our MA images are obtained from a small group of patients (28 eyes from 20 subjects) and we also do not intend to perform patient-specific studies, the AOSLO images are split in a random fashion, meaning that images from one subject may belong to either training dataset or testing dataset or both. We perform a 10-fold cross-validation using these four folds of images, meaning that these images are further separated into 10 folds, with nine folds used for training after augmentation and one fold used for validation. Overall, the split of the original dataset (87 images) for training, validation and testing 63:7:17. The original training images are augmented to 63 × 32 = 2016 to improve the training performance.

The loss function is a combination of binary cross-entropy and Dice loss,

$$\begin{array}{c} Loss=BCE+\alpha \times Dice, \end{array}$$

where BCE and Dice are defined as

$$\begin{array}{c} \begin{array}{c} BCE=\sum_{n}\left[y_{n}\cdot lnx_{n}+\left(1-y_{n}\right)*\ln\left(1-x_{n}\right)\right]\\ DICE=\frac{2\left|X\cap Y\right|}{\left|X\right|+\left|Y\right|} \end{array} \end{array}$$

Here, *X, Y* are model prediction and target, respectively, *xn, yn* are pixel values in *X* and *Y*; *α* is set to 0.2 to equalize the contribution of BCE and Dice to the loss. We also introduced the Hausdorff distance following the definition from Karimi and Salcudean,^43^ which is commonly used to incorporate the contour difference of two shapes, into the loss function, but it did not improve our segmentation results (Supplementary Fig. S6).

We use the Adam optimizer with learning rate of 0.001 and weight decay of 10^−^^8^. During training, a Plateau scheduler is specified such that once the validation loss does not decrease for five epochs, the learning rate is decreased to 1*/*10 of the current value to facilitate the convergence of the AOSLO-net. The training is initially set to last 200 epochs, but it may end earlier because of the implementation of Plateau scheduler. The batch size is 16, and the image resolution is 512 × 512 × 2 (two channels are enhanced AOSLO images and perfusion map).

### Postprocessing and Ensembling

#### Binarization

The pixel value of the output images from AOSLO-net lies within the range (0, 1) resulting from using the sigmoid activation function in the last layer of AOSLO-net. To quantify the geometries of MAs, we convert these pixel values into a binary form, meaning that the pixel value is either 1 (belongs to an MA), or 0 (not MA). A threshold of 0.5 is applied to binarize the segmented images in the current study.

#### Clearing

We note that some segmented images contain small fragments that are mistakenly predicted as MAs. Thus we specify an area threshold of 1024 pixels, below which the fragments are removed from the segmented images.

#### Ensembling

Following the work of Isensee et al.,^38^ we use the ensembling method by selecting the three best models, of the 10 trained models, based on their performance on the validation set and perform a union of their outputs to improve the model performance. Some examples illustrating the effect of postprocessing are shown in Supplementary Figure S8.

### Performance Metrics

We evaluate the segmentation performance of AOSLO-net and other segmentation models using the Dice coefficient and intersection over union (IoU), which is defined as

(1) $$\begin{array}{c} IoU=\frac{\left|X\cap Y\right|}{\left|X\cup Y\right|},\; \end{array}$$

where *X* and *Y* denote the matrix representations of the target image and the corresponding prediction image, respectively.

### Geometric Metrics

The quantitative evaluation of the morphologies of MAs focuses on two geometric indexes, namely, the diameter of the MA body (LC) and the narrowest width of its parenting vessels (NC). These two indexes can be used to calculate the body-to-neck ratio (BNR = LC/NC) of MAs, a metric that is associated with probability of MA for rupture and thrombosis.^15^^,^^20^^,^^44^

## Results

### Morphology of MA Body and Its Feeding and Draining Vessels

**Figure 3.**
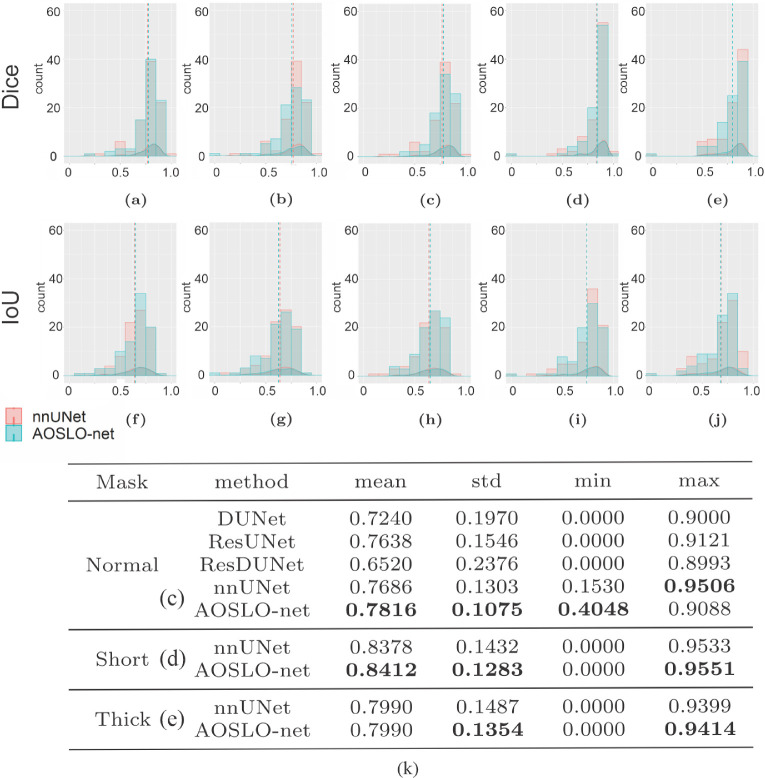
Performance metrics of AOSLO-net and nnUNet as well as other deep-learning models on segmenting the AOSLO images. Histogram and density distribution of Dice score (a–e) and IoU (f–j) obtained from AOSLO-net and nnUNet when they are trained using (a, f) perfusion map and normal mask set, (b, g) enhanced AOSLO images and normal mask set, (c,h) two-channel MA images and normal mask set, (d, i) two-channel MA images and short mask set, (e, j) two-channel MA images and thick mask set. The result of AOSLO-net is denoted by cyan color whereas the result of nnUNet is denoted by red color. The dash lines denote the mean values of the Dice or IoU distributions. (k) The overall performance (mean value), performance stability (standard deviation), the worst and best case performance (minimum and maximum value) of Dice score of different AI models in segmenting AOSLO images.

We first train the AOSLO-net and nnUNet using perfusion map (Fig. 1c), enhanced AOSLO (Fig. 1d) and two-channel images (Fig. 1e), respectively, to examine which image modality can optimize the model performance. The normal mask set (Fig. 1f) is used as the target in these training processes. Our results in Figures 3a–c and 3f–h and Supplementary Figure S3 show that higher Dice and IoU scores are achieved for both nnUNet and AOSLO-net models when two-channel images are used, suggesting that two-channel images could provide more MA geometrical information to the segmentation models than the other two image modalities. Therefore, in the following section, we use the two-channel image set as the input of segmentation models to assess the model performance on different mask sets, including normal mask set, short mask set and thick mask set. The performance of AOSLO-net and nnUNet on these three mask sets is summarized in Figure 3, which shows that based on Dice and IoU, AOSLO-net achieves more high-quality predictions and fewer low-quality predictions for these three groups of masks. Specifically, when models are trained with the normal and short mask sets, as shown in Figures 3c and 3d, AOSLO-net achieves mean Dice scores of 0.7816 and 0.8412, respectively, which are higher than 0.7686 and 0.8378 of nnUNet. Additionally, the performance of AOSLO-net appears to be more stable than nnUNet, given smaller standard deviations in the results of AOSLO-net. When these two models are trained with a thick mask set (Fig. 3e), although the mean Dice scores of AOSLO-net and nnUNet are the same, AOSLO-net preserves a stable performance with a relatively smaller standard deviation in Dice score. In addition to nnUNet, as listed in Supplementary Figure S4, we specifically compare the performance of AOSLO-net with other popular CNN-based models (i.e., Deformable UNet,^32^ ResUNet^45^ and Deformable ResUNet,^45^^,^^46^) using the normal mask dataset. The statistics of the Dice scores listed in Figure 3k suggest that AOSLO-net outperforms these models significantly in mean Dice score and standard deviation.

**Figure 4.**
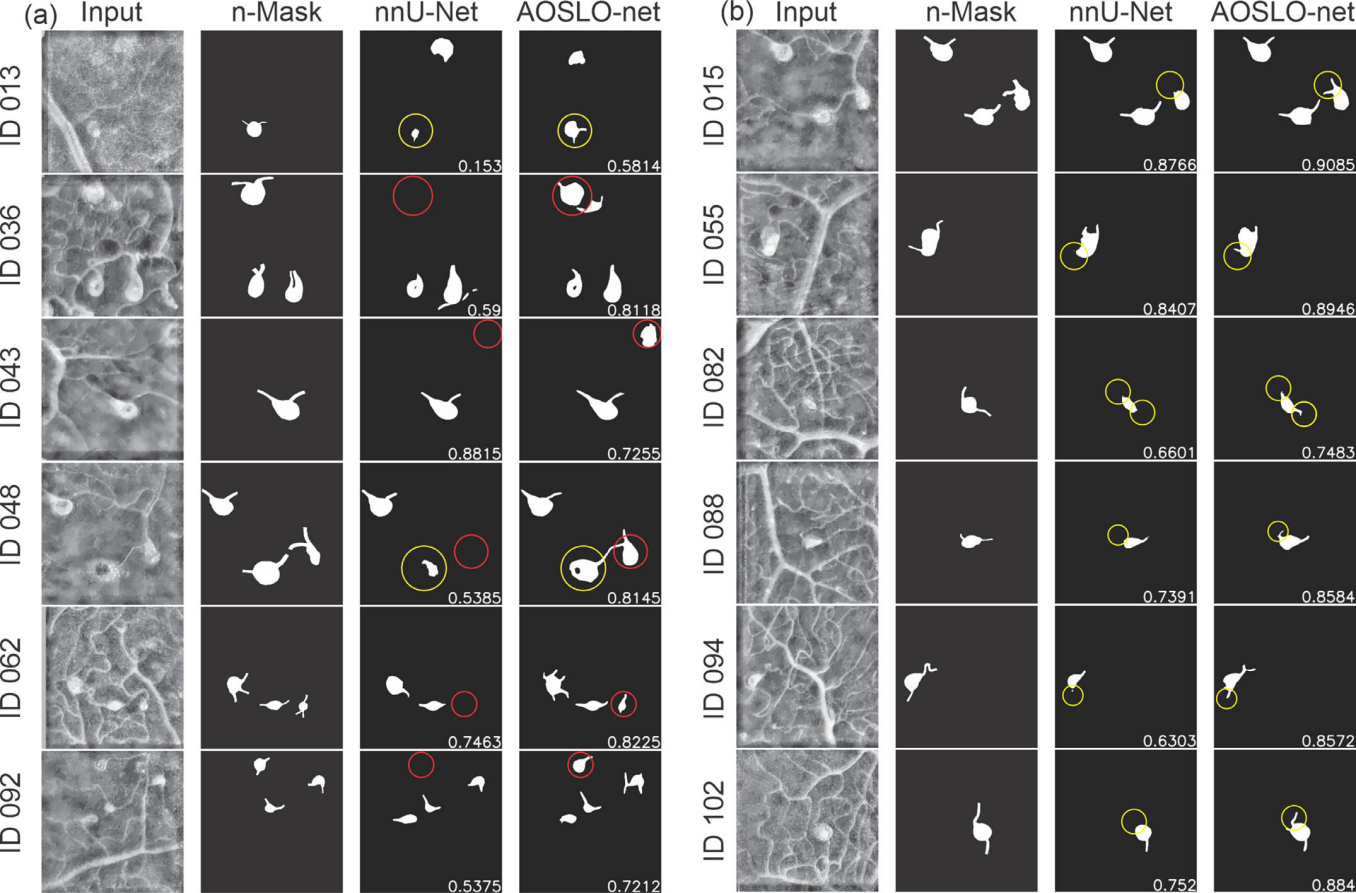
Typical examples of AOSLO-net outperforming nnUNet on detecting (a) the MA body and (b) parenting vessels of MAs when they are trained using normal mask set (n-Mask). *First column*: enhanced AOSLO images. *Second column*: normal masks used to train the models. *Third column*: segmentation results of nnUNet. *Fourth column*: segmentation results of AOSLO-net. Numbers in images are the Dice scores. (a) For images with IDs 036, 043, 048, 062, and 092, the AOSLO-net is able to detect MAs, which are missed by nnUNet (marked in *red circles*). For images with IDs 013 and 048, the AOSLO-net can better reconstruct the full shape of MAs compared to those of nnUNet (marked in *yellow circles*). We also note that in cases of IDs 013, 043 and 092, the segmentation models can even detect some potential MAs, which are not marked in the masks. (b) For images with IDs 015, 055, 082, 088, 094, and 102, the AOSLO-net is capable of detecting both feeding and draining vessels connected to the MAs, whereas the nnUNet may miss one or both of the parenting vessels (marked in *yellow circles*). These results show that the AOSLO-net is more reliable in detecting MA parenting vessels from the input images, which are essential in the MA classification—a downstream task for disease diagnosis.

To further examine the capabilities of AOSLO-net and nnUNet in extracting the detailed MA features, we perform image-wise analysis by comparing individual pairs of masks and model predictions. We focus on the model performance on detecting MA bodies and their feeding and draining vessels, respectively. Typical examples of the comparisons between nnUNet and AOSLO-net on detecting MA bodies are illustrated in Figure 4a. We note that AOSLO-net can identify the targeted MAs and extract MA bodies that are comparable with the masks, particularly for images containing multiple MAs, like IDs 036, 048, 062 and 092. In contrast, nnUNet fails to detect some of the MAs (highlighted in red circle). As for the vessel detection, Figure 4b shows that nnUNet may ignore one or both of the parenting vessels of MAs (highlighted in yellow circle), whereas AOSLO-net can segment out these missed vessels (from complicated background with numerous vessels), which is essential for further morphological analysis. Quantitatively, there are 11  images in which AOSLO-net can detect vessels that nnUNet cannot detect and eight images in which AOSLO-net can detect MAs that nnUNet cannot detect. On the other side, there are only two images in which nnUNet can detect vessels that AOSLO-net cannot and two images in which nnUNet can detect vessels that AOSLO-net cannot.

**Figure 5.**
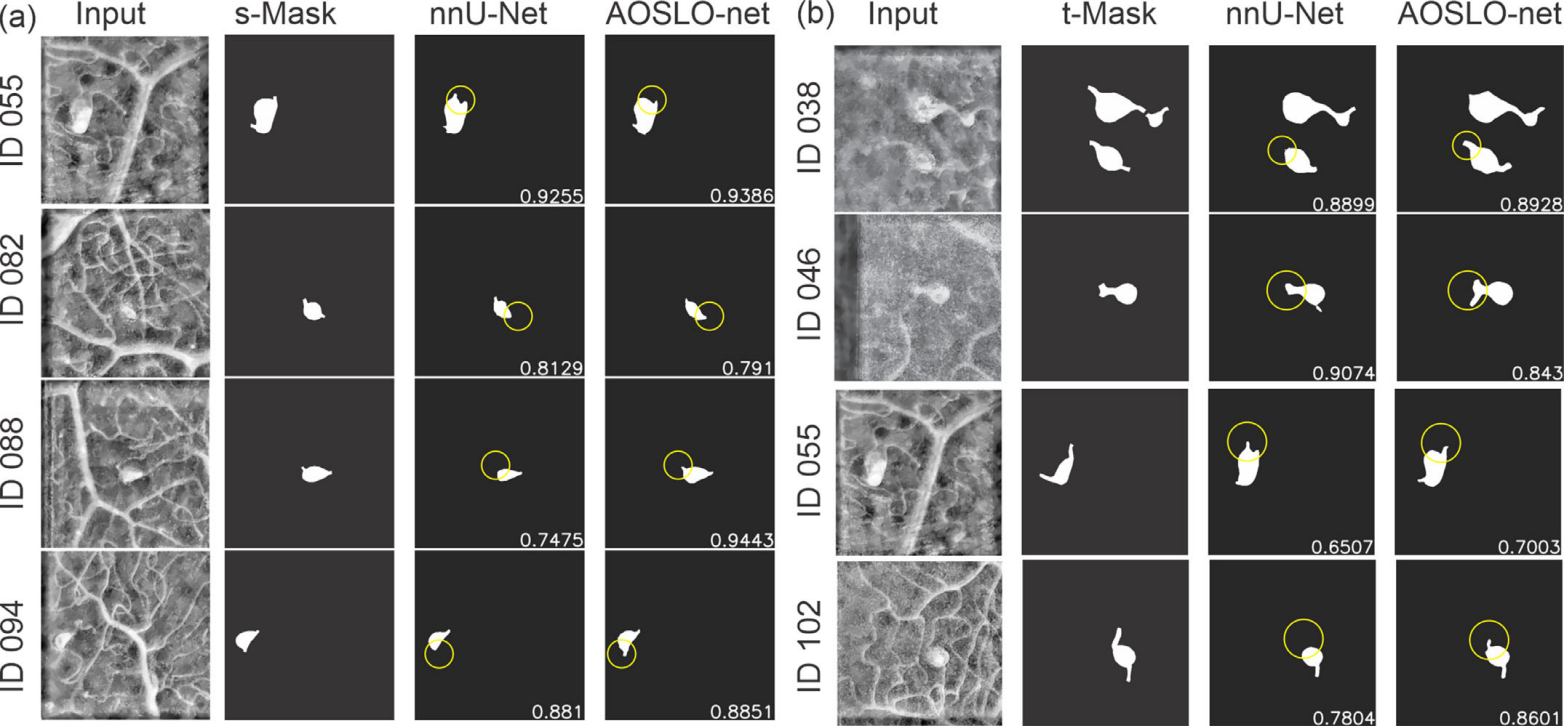
Typical examples of AOSLO-net outperforming nnUNet on detecting the parenting vessels of MAs when they are trained using (a) short mask set and (b) thick mask set. *First column*: enhanced AOSLO images. *Second column*: normal masks used to train the models. *Third column*: segmentation results of nnUNet. *Fourth column*: segmentation results of AOSLO-net. Numbers in images are the Dice scores. (a) AOSLO-net are capable of detecting the parenting vessel of MAs in images with ID 055, 082, 088 and 094, whereas nnUNet misses one of the parenting vessels(marked in *yellow circles*). Particularly, nnUNet again mistakenly predicts the vessel on the upper left for the case of ID 055. (b) AOSLO-net are capable of detecting the parenting vessel of MAs in images with ID 038, 046, 055 and 102, whereas nnUNet misses one of the parenting vessels(marked in *yellow circles*). In case of ID 055, implementation of thick mask set improves the segmentation results of AOSLO-net, but not for nnUNet.

Next, we use another two sets of masks: one with shorter vessels compared to the normal dataset and one with thicker vessels, as learning targets to train AOSLO-net and nnUNet and compare their performance on detecting the feeding and draining vessels of MAs. We first train the model with short vessel mask dataset. The predictions from the two models in Figure 5a show that the performances of nnUNet and AOSLO-net on detecting feeding and draining vessels are both compromised, as the shorter vessel masks provide less vessel end information. However, the AOSLO-net still can detect correct vessels in these four cases whereas nnUNet fails to predict some of the vessel ends (highlighted in yellow circle). There are three images in which AOSLO-net can detect vessels that nnUNet cannot detect and seven images in which AOSLO-net can detect MAs that nnUNet cannot detect. On the other hand, there is no image in which nnUNet can detect vessels that AOSLO-net cannot, and no image in which nnUNet can detect vessels that AOSLO-net cannot. These comparisons indicate that AOSLO-net is more robust against vessel length of the training masks.

When trained with thick vessel mask set, as shown in Figure 5b, AOSLO-net outperforms the nnUNet although the predictions of nnUNet have improved in detecting the feeding and draining vessels of MAs. Figure 5b shows that although nnUNet misses the MA bodies and vessel ends connected to MAs (highlighted in yellow circle), AOSLO-net can detect these feeding and draining vessels. When trained with thick masks, there are 11 images in which AOSLO-net can detect vessels that nnUNet cannot detect, and five images in which AOSLO-net can detect MAs that nnUNet cannot detect. On the other hand, there are only five images in which nnUNet can detect vessels that AOSLO-net cannot, and no image in which nnUNet can detect vessels that AOSLO-net cannot. These results again demonstrate the robustness of AOSLO-net when trained with varying vessel thickness.

### Morphology Quantification for the MA Segmentation Maps

**Figure 6.**
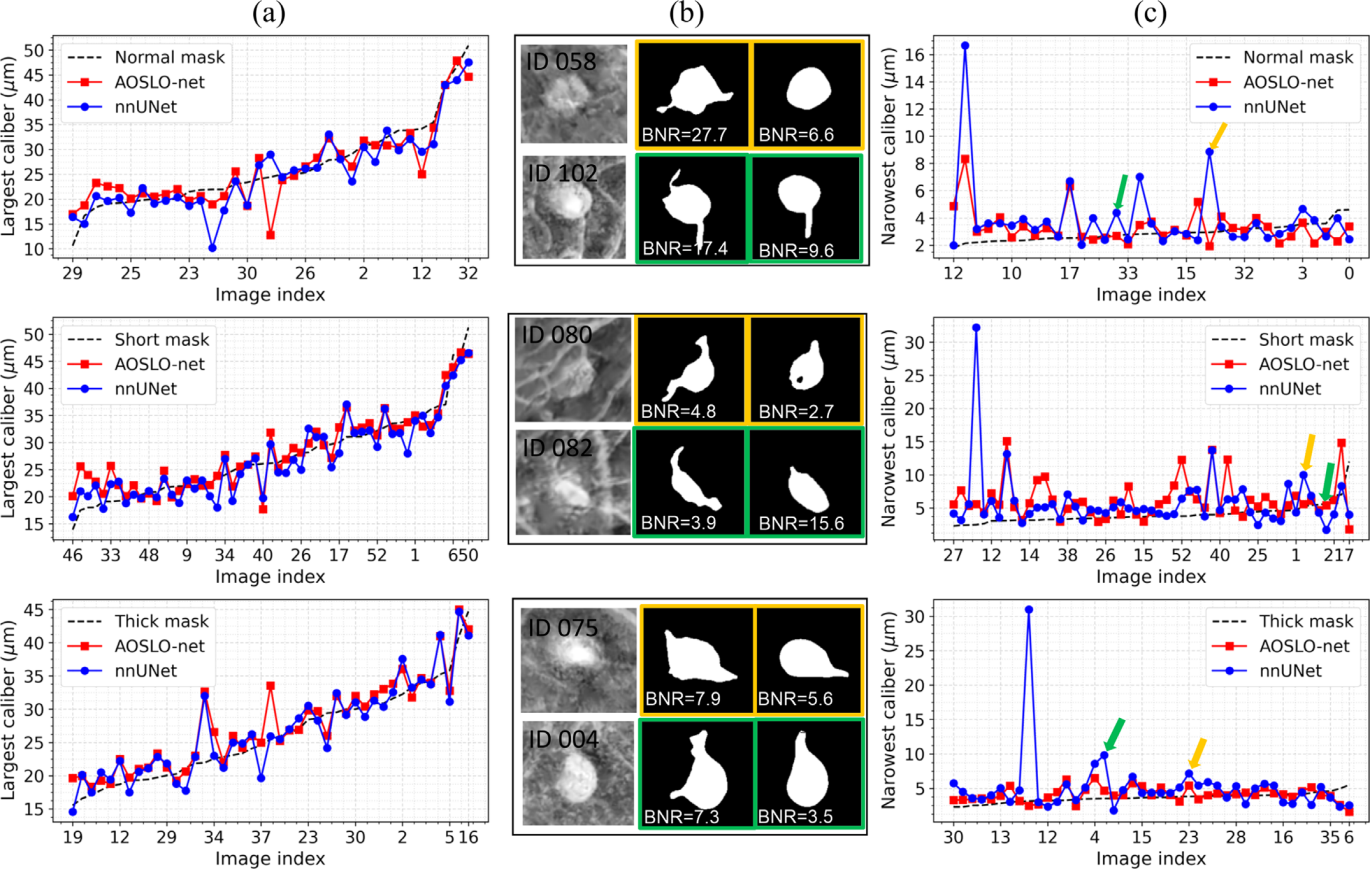
Quantification results for the largest caliber and narrowest caliber factors for the segmented MAs predicted by AOSLO-net and nnUNet trained with three sets of MA masks (normal, short and thick masks). (a) The largest caliber (LC) results for the segmented MAs. The *black dashed lines* represent the reference MA LC values for different MAs, whereas the *red* and *blue* curves are the MA LC quantification results based on the segmentation maps from AOSLO- net and nnUNet using three different masks (from top to the bottom row). (b) Examples for the original enhanced perfusion maps (*first* *column*), segmented MAs using AOSLO-net (*second* *column*), and nnUNet (*third* *column*) trained with different MA masks. The MA segmentation results in *yellow* and *green bounding boxes* correspond to the images in (c) marked with *yellow* and *green arrows*. (c) Narrowest caliber (NC) quantification results for segmented MAs using AOSLO- net and nnUNet trained with three different MA masks; the *black dashed lines* represent the reference MA NC values computed using the original three types of MA masks; the *red* and *blue curves* represent the NC quantification results using segmentation maps obtained by AOSLO- net and nnUNet using three different masks (from top to the bottom row). Quantification of MA shapes using MAs segmented from AOSLO-net results in smaller discrepancy between the model predictions and groundtruth (*dotted lines*) due to the better performance of AOSLO-net in segmenting the parenting vessels of MAs.

To quantify the shape of the segmented MAs, we compute three important MA morphological indexes (“largest caliber” [*LC*], “narrowest caliber” [*NC*], and the “body-to-neck ratio” [*BNR*]) as defined in.^15^ Specifically, we first compute the MA skeleton (or medial-axis) for every single MA using the “Scikit-image” package,^47^ and then apply the Euclidean distance transformation to compute the medial radius distances *D* = {*di*|*i* = 1*,* 2*, ..., N* } (*N* is the number of points on the MA skeleton) from all points of the MA skeleton to the background pixels (i.e., pixels of each MA contour). Consequently, the *LC* value for each single MA corresponds to twice of the largest distance value in the sorted distance list *D_sorted_*. Due to the varied vessel lengths of different MAs, we calculate the NC value for each single MA by selecting the 10 smallest medial radius distances from *D_sorted_*, and double the average medial radius distances as the final *NC* value. Based on the *LC* and *NC* values, the BNR value for each MA can be computed by using *LC/NC*. Figure 6 gives some examples of the LC and NC quantification results for the segmented MAs predicted by AOSLO-net and nnUNet trained with three different MA masks (normal, short and thick mask sets). From Figures 6a and 6c, we can find that the AOSLO-net trained with thick MA ground truth masks attains the best MA segmentation performance; the NC quantification results (red curve) as shown in the third row of Figure 6c are very close to the reference NC values (black dashed line) obtained from the thick masks. More details about the corresponding examples of the enhanced MA perfusion maps and the MA segmentation results using AOSLO-net and nnUNet are, respectively, shown in columns 1 to 3 of Figure 6b. AOSLO-net can effectively detect the important small vessels for the heterogeneous MAs (see the second column in Figure 6b), which play a very important role in different downstream tasks, (e.g., MA morphological parameter quantification [NC, BNR, convexity], MA severity stratification, and in hemodynamics simulations). The three blue dots with very high NC values in different rows of Figure 6c are the same MAs for which nnUNet fails to detect the small vessels.

Quantification of the mean square error for the predictions of the NC and LC of MAs are summarized in the Table, which shows that AOSLO-net outperforms the nnUNet for the three groups of test datasets, and the short mask set is more likely to lead to erroneous results.

**Table.** The Mean Squared Errors of the Predictions From AOSLO-Net and nnUNet for the LC and NC Values of MAs Based on Normal, Short, and Thick Make Sets

	LC		NC	
Mask Type	AOSLO-Net	nnUNet	AOSLO-Net	nnUNet
Normal	12.11	13.39	2.56	8.77
Short	8.56	9.86	15.86	25.64
Thick	8.62	9.65	1.73	22.06

## Discussion

Although two-dimensional fundus photography has been primarily used for DR screening and severity grading, other advanced imaging modalities, such as AOSLO, which is currently used for disease investigation and primarily for research purposes, can provide additional information regarding the retinal microvascular pathology, such as monitoring of variations in blood flow rates,^48^ detection and identification of the MA morphologies,^14^ enhanced visualization of MA thrombus status,^15^ and more. The information from this imaging technique could be potentially used not only to improve the future accuracy of DR screening but also to better predict the rate of DR worsening, with the goal of eventually providing individualized management and treatment intervention plans.^49^ Because of the rapidly rising global prevalence of diabetes and shortage of skilled graders for retinal images, implementation of automatic screening techniques is desirable to accommodate the corresponding increasing need to screen and evaluate patients with diabetes ocular complications.^25^ Development of an automated segmentation technique for AOSLO images, which are at much smaller scale than fundus photographs, represents a unique challenge and provides the opportunity to elucidate the steps of in vivo worsening and regression of MAs, a key lesion in DR that is associated with surrounding neuroretinal pathology and visual function in diabetes.^23^

In the last decade, DCNN has become the prominent technique in diagnosing DR through analysing the clinical features on retinal fundus images. However, our study shows that various DCNN models, including the state-of-art segmentation model, nnUNet,^38^ cannot segment MAs from the raw AOSLO images at a quality that is sufficient for further quantification of their geometrical characteristics because of the following issues with the AOSLO images: (1) the large size imbalance between the MA bodies and their parenting vessels for some of the MAs; (2) the sizes of some background vessels are similar to the sizes of MAs; and (3) blurred MA boundaries due to eye movement during the imaging process.

In this work, we develop the first AOSLO-net, a specially designed DCNN model for automatic segmentation of MA bodies along with their parenting vessels from AOSLO images. AOSLO-net is composed of a deep network to capture and extract the high-level geometric features of MAs.

The specific contributions of this work are as follows:

• Input multimodality: Based on the specific features of AOSLO images, we first generate a two-channel image dataset as model inputs by concatenating the raw AOSLO images, which give a better description of the boundaries of MA bodies, with perfusion maps that highlight the blood flow traveling through the MAs for detecting the MA's parenting vessels.
• Data preprocessing: We design a denoise-enhance framework, including a nonlocal mean denoise method to eliminate the background noise and CLAHE to enhance the contrast, to improve the quality of the images before they are used to train the network. Although these methods are known in the literature, the combination we proposed here is original, to the best of our knowledge.
• Data postprocessing: We use 10 random initializations of AOSLO-net to obtain 10 outputs. Then, we take the union of the best models (selected from validation) to create final predictions for test dataset; we found that this increases the likelihood of capturing all the potential MAs and their parenting vessels without obtaining many false-positive predictions.

Equipped with these techniques, our results show that AOSLO-net can detect the detailed geometrical features of MAs (e.g., the feeding and draining vessels of Mas), and it outperforms nnUNet^38^ and other classic segmentation models, such as Deformable UNet,^32^ ResUNet,^45^^,^^50^ and Deformable ResUNet,^45^^,^^46^^,^^50^ which have demonstrated high accuracy in detecting different features, such as retinal vessels, hemorrhage, and MAs, from fundus images.

Because AOSLO imaging is not a standard technique to screen for DR, our AOSLO dataset contains less than 100 images, which, to the best of our knowledge, is still the largest available AOSLO dataset for MA detection. The limited size of the dataset may cause overfitting in the model training process because we use a deep structure to capture the detailed structure of MAs and their parenting vessels. Thus we augment the training data by flipping, rotating, scaling the original image to increase the diversity of data available for model training. We also note that MAs with different shapes are detected from these AOSLO images, but there is an imbalance in the number of images in different types of MAs. This requires the AOSLO segmentation to be a composition of many few-shot problems. Therefore data augmentation is necessary to train the deep network and fully utilize the data. To optimize the data augmentation, we use horizontal and vertical flip, rotation in uniformly distributed angles and scaling to achieve maximum space configuration of MAs. As a result, the AOSLO-net can learn MA geometric information very effectively from the limited dataset. We note that augmentation techniques cannot recover all the missing regions of the statistical distribution of the dataset. Thus the good performance on the dataset may not guarantee similar performance on new images with different stochastic properties. Because our data base at the Joslin Diabetes Center is constantly expanding, we plan to use the transfer learning method to continuously refine the model as new AOSLO images become available. In future work, we will report on an automatic classification system based on the AOSLO-net.

The relationship between model performance metrics and morphological features of MAs is also investigated carefully in our work. Accurate detection of feeding and draining vessels of MAs is critical to determine MA morphology, which is important to predict blood flow characteristics and estimate the likelihood of thrombosis within the MA^14^ and explain the mechanism of MA turnover.^51^ Because the areas of the end of the parenting vessels are much smaller compared to the areas of the whole MAs, regional loss, such as Dice and IoU, possibly ignore these vessel ends and place too much emphasis on the MA bodies. However, correct segmentation of MA bodies alone is not sufficient for further classification. Thus metrics such as Dice and IoU are important but should not be the only metrics to evaluate the performance of MA segmentation. Our results demonstrate that AOSLO-net not only achieves high scores on these two metrics but also captures the detailed features of the feeding and draining vessels of MAs.

We note that the size of our AOSLO image dataset is limited to 87 images, although it is still the largest one available in the literature to the best of our knowledge. With the implementation of data augmentation techniques and pretraining using Imagenet, we have been able to train the AOSLO-net with this dataset. However, we are not able to directly train a classifier to categorise these MAs into different subgroups (e.g., focal bulging, saccular, fusiform, mixed saccular/fusiform, pedunculated and irregular-shaped MAs^14^) because the number of MAs in these subgroups is highly imbalanced. In particular, mixed saccular/fusiform, pedunculated and irregular-shaped MAs are rarely observed in our dataset. Thus we focus on MA segmentation and quantify their geometrical indexes, which can be used as metrics for MA classification. Our coauthors at Joslin Diabetes Center, the world's largest diabetes research center and diabetes clinic, will continue to collect AOSLO images from diabetic patients to supply a growing, robust dataset that is available to the public for performing MA classification and future model refinement.

In summary, we present AOSLO-net, a deep neural network framework with customized training policies to automatically segment MAs from AOSLO images. The validity of the model is demonstrated by the good agreement between the predictions of our AOSLO-net model on 87 patient-specific MA images and the MA masks generated by ophthalmologists and skillful trainees. In particular, our results suggest that AOSLO-net can accurately capture the detailed features of MAs, such as their parenting vessels, which is essential for the MA shape classification. We also show that the performance of AOSLO-net exceeds existing segmentation models, thereby promising to be a more effective MA segmentation method in the clinic. Moreover, AOSLO-net can be potentially used as a new tool to analyze the pathology of MAs and improve the current understanding of microvascular pathology in DR. As the first attempt for automatic segmentation of retinal microaneurysms from AOSLO images, this work can potentially motivate and steer the development of new models in this area and shift the attention of the community from the conventional fundus images to more advanced imaging modalities.

## Supplementary Material

Supplement 1
