# Supplementary material for: AOSLO-net: A Deep Learning-Based Method for Automatic Segmentation of Retinal Microaneurysms From Adaptive Optics Scanning Laser Ophthalmoscopy Images
Source: Transl Vis Sci Technol. 2022 Aug 8;11(8):7. doi: 10.1167/tvst.11.8.7 (PMC9366726; doi:10.1167/tvst.11.8.7)
Supplement: Supplement 1 [file tvst-11-8-7_s001.pdf]

Supplementary Information: AOSLO-net: A deep learning-based method for automatic segmentation of retinal microaneurysms from adaptive optics scanning laser ophthalmoscope images

Qian Zhang*[1], Konstantina Sampani*[2,3], Mengjia Xu*[1,5], Shengze Cai[1], Yixiang Deng[5], He Li[1], Jennifer Sun†[2,4], George Em Karniadakis†[1,6]

[1]Division of Applied Mathematics, Brown University, Providence, RI 02912, USA
[2]Beetham Eye Institute, Joslin Diabetes Center, Boston, Massachusetts, USA
[3]Department of Medicine, Harvard Medical School, Boston, Massachusetts, USA
[4]Department of Ophthalmology, Harvard Medical School, Boston, Massachusetts, USA
[5]McGovern Institute for Brain Research, Massachusetts Institute of Technology, Cambridge, MA 02139, USA
[6]School of Engineering, Brown University, Providence, RI 02912, USA

* These authors contributed equally to this work.
† To whom correspondence should be addressed.
E-mail: george_karniadakis@brown.edu, Jennifer.Sun@joslin.harvard.edu.

1 Data

1.1 Preprocessing on Perfusion Map

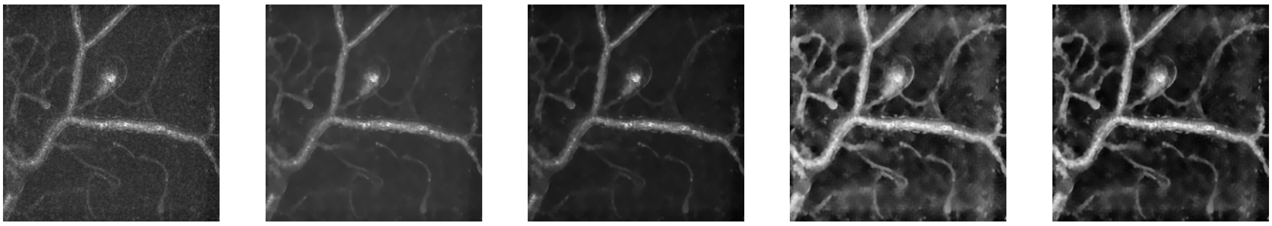

(a) Perfusion Map **(b)** Denoise **(c)** Normalization **(d)** CLAHE **(e)** Gamma

Figure 1. An example of the sequential image preprocessing steps for perfusion map. (a) Raw perfusion map, which contains background noise and suffers from low image contrast. **(b)** Perfusion map after applying the non-local means filtering method, which creates a denoised image. **(c)** Perfusion map after intensity normalization in the range of $[0, 1]$, which enhances the contrast. **(d)** Perfusion map after employing contrast limited adaptive histogram equalization (CLAHE). **(e)** Perfusion map after employing Gamma correction. We applied these sequential preprocessing steps for all the perfusion maps in AOSLO-net training and testing.

1.2 Data augmentation

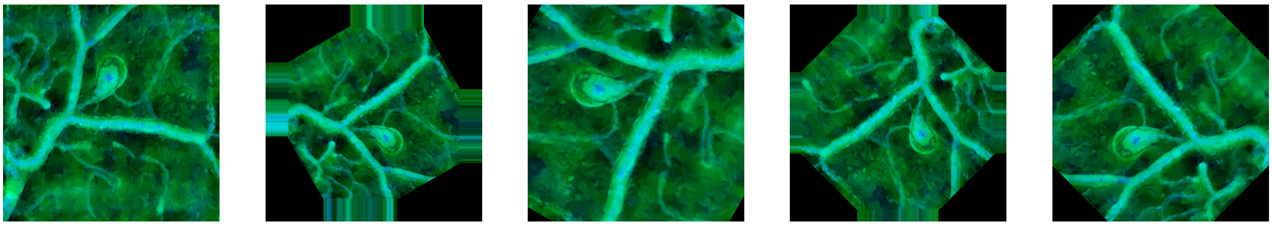

(a) (b) (c) (d) (e)

Figure 2. Examples of data augmentation. (a) Two-modality image (using the processed perfusion map and the enhanced AOSLO image as two channels in RGB space). **(b)-(e)** Augmented images from (a) by performing different transformations: **(b)** flipping vertically, rotating with an angle $\theta = \pi/6$ and scaling with a factor $\lambda < 1$; **(c)** flipping horizontally, $\theta = \pi/3$ and $\lambda > 1$; **(d)** flipping both horizentally and vertically, $\theta = \pi/4$ and $\lambda < 1$; **(e)** no flipping, $\theta = 3\pi/4$ and $\lambda \sim 1$. The rotating angle was uniformly distributed, while the scaling factor was randomly selected in an appropriate range when we performed data augmentation on our AOSLO dataset.

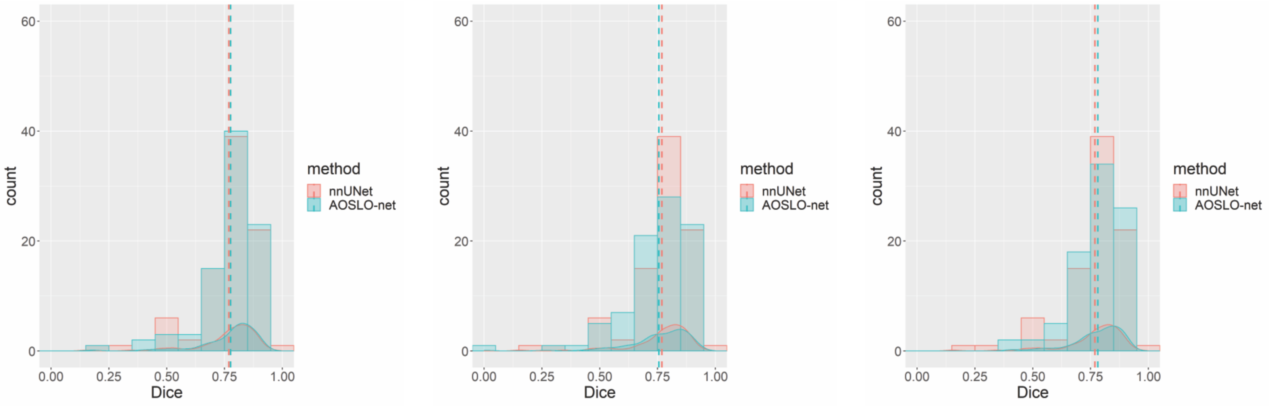

(a) (b) (c)

Modality	mean	std	min	max
(a) Perfusion Map	0.7762	0.1251	0.1791	0.9216
(b) Enhanced AOSLO image	0.7554	0.1458	0.0000	0.9222
(c) Both	0.7816	0.1076	0.4048	0.9088

(d)

Figure 3. Performance of AOSLO-net and nnU-Net with different input modalities. (a) Histogram and density distribution of Dice score obtained by using the perfusion maps as input. The result of AOSLO-net is denoted by cyan color whereas the result of nnU-Net is denoted as red color. The mean Dice score (denoted by dash line) of AOSLO-net is slightly higher than that of nnU-Net. **(b)** Histogram and density distribution of Dice score obtained by using the enhanced images as input. In this case, the mean Dice score of AOSLO-net is slightly lower than that of nnU-Net. Moreover, the AOSLO-net is more likely to make low-quality predictions. **(c)** Histogram and density distribution of Dice score obtained by using two-modality images as input, where the AOSLO-net performs better than the nnU-Net. **(d)** The statistics of AOSLO-net based on different input modalities. Using two-modality images as input results in the best performance.

2 AOSLO-net model

2.1 Network

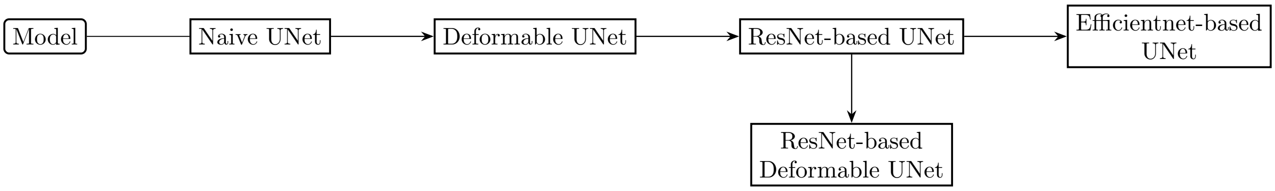

Figure 4. A list of segmentation models that have been tested in the current work. The arrow points to the model with a better performance in terms of Dice and IoU score.

UNet. The vanilla version of UNet [1] was proposed in 2015. This network tends to erroneously predict the background vessels as MAs, and overall the predicted MA shapes are not satisfactory.
Deformable UNet. Deformable UNet [2] uses the deformable convolution layer instead of the original convolution layer. Deformable UNet shows improved performance on distinguishing the MAs from the vessels, but it fails to capture the MA shape and vessels connected to MAs.
ResUNet. We implemented ResUNet with `Segmentation Models Pytorch` [3], a python package. ResNet101 was used as backbone, which included more parameters than the official implementation. As a result, this UNet is able to better capture the MA shape and the inlet outlet vessels. However, the overall performance of this UNet is not sufficient for MA shape quantification.
ResNet-based Deformable UNet. The main body of this kind of UNet is the same as the ResNet-based UNet, but the convolution layers are replaced to be deformable ones. We implemented it by substituting ordinary convolution kernel in `Segmentation Models Pytorch` with a deformable convolution kernel [4]. But these modifications do not improve model performance on segmenting AOSLO images.
nnUNet. nnUNet [5] is an automatic medical image segmentation tool. It computes pipeline fingerprint from dataset features, and use this to design UNet structure for segmentation. It is data efficient and has a good generalization capability.
EfficientNet-based UNet. Using EfficientNet-b3 [6], a SOTA classification network, as encoder in AOSLO-net has show significant improvements over the models listed above. We use the implementation from `Segmentation Models Pytorch`.

2.2 Loss

Binary CrossEntropy (BCE) is the most common loss function for classification and segmentation. However, in our study, there is also a strong imbalance since the MA areas are relatively small compared with the whole image. Under these circumstances, the BCE loss was overwhelmed and the neural network predicted MAs as background. To overcome this imbalance, we use regional loss based on Dice and IoU to maximize the intersection ratio of the prediction and the ground truth.

Despite the advantage of regional loss like Dice and IoU, they may cause oscillation of the training loss curve. Thus, we added BCE as a smoothing loss to the regional loss. We used a coefficient $\alpha = 0.2$ to equalize contribution of BCE and regional loss as shown in Figure 5.

We also tested the Hausdorff distance, which is defined as

$$d_H(X,Y) = \max\{\sup_{x\in X} d(x,Y), \sup_{y\in Y} d(y,X)\}, \tag{1}$$

where X is prediction and Y is target, d is metric of the space, which is the Eucleadian distance in our case. The Hausdorff distance is used to describe the contour difference of two shape. However, as shown in Fig. 6, the effect of using the Hausdorff distance as part of the loss was not notable to our current method.

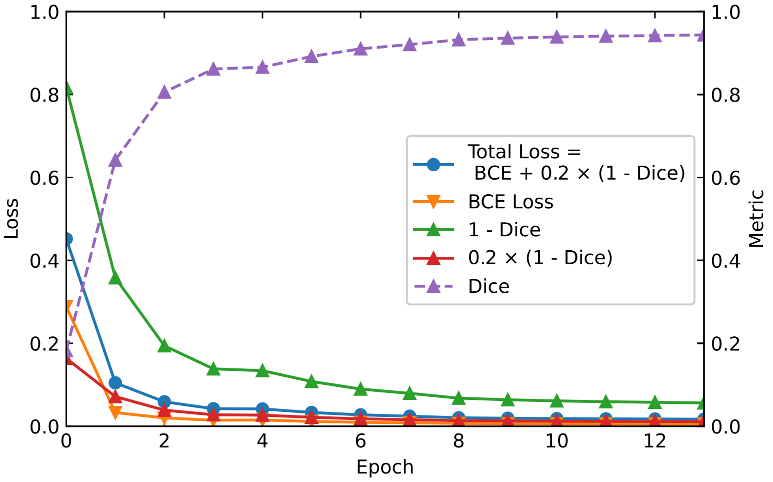

Figure 5. Training Losses and Dice score of AOSLO-net versus training epochs. The original Dice loss (in green color), which is defined as $1 - \text{Dice}$, is much larger compared to the binary cross entropy (BCE) loss (in orange). In order to balance the contribution of two terms in the total loss function, a weighting coefficient 0.2 was applied for the Dice loss. The convergence of the rescaled Dice loss, $0.2 \times (1 - \text{Dice})$, is shown in red color, while the total loss is shown in blue.

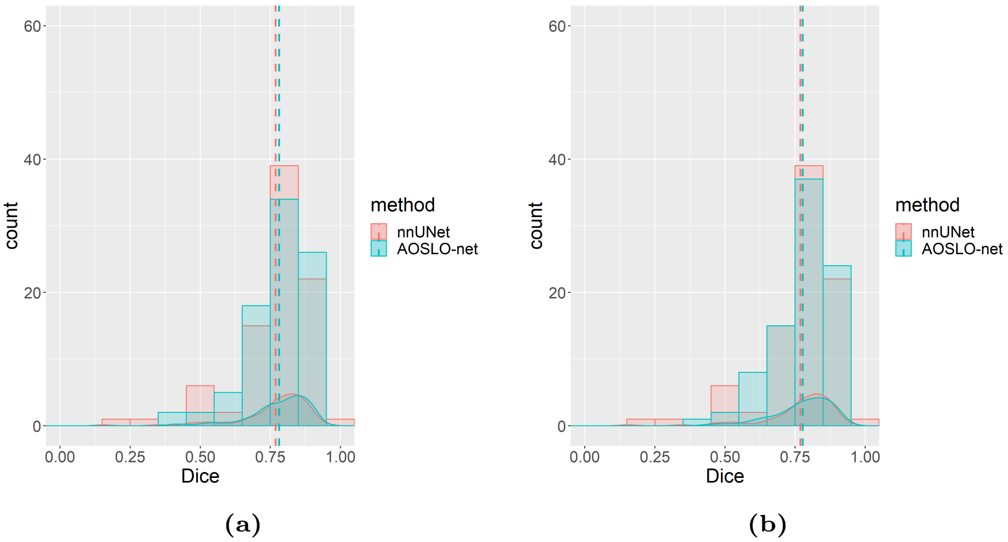

Figure 6. Performance of AOSLO-net and nnU-Net with and without Hausdorff distance loss. (a) Histogram and density distribution of Dice score when the models are trained with Hausdorff distance as part of the loss. **(b)** Histogram and density distribution of Dice score when the models are trained without using the Hausdorff distance. We observe that applying the Hausdorff distance did not improve the performace of AOSLO-net. Instead, the mean value of Dice score dropped from 0.7816 to 0.7774 for the AOSLO-net.

2.3 Optimizer

We first used the SGD as optimizer, the default for many deep learning based computer vision problems and we found that loss descending rate is very slow. Thus, we employ Adam optimizer and the training usually converged in 20 epochs. More importantly, after using Adam as optimizer, the segmentation performance on validation and test set is also improved.

In order to prevent the overfitting, we used the Plateau learning rate scheduler. When the validation loss does not decrease for 5 epochs, the scheduler decreases the learning rate to 1/10 of the current one, so the network converges before overfitting. The effect of using the scheduler is illustrated in Figure 7, where the training finished in less than 15 epochs, while nnU-Net will use 1000 epochs by default.

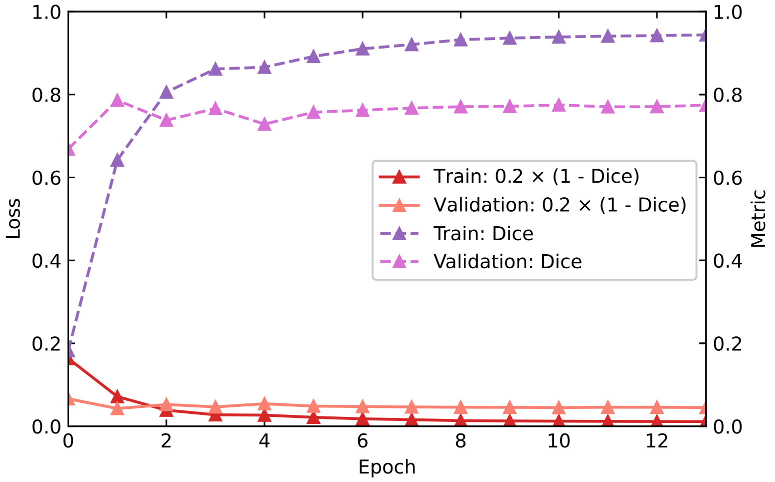

Figure 7. Loss and Dice score of AOSLO-net in training and validation. The AOSLO-net undergoes a quick convergence and achieves a good validation Dice score upon convergence.

3 Post Processing

3.1 Binarization and Clearing

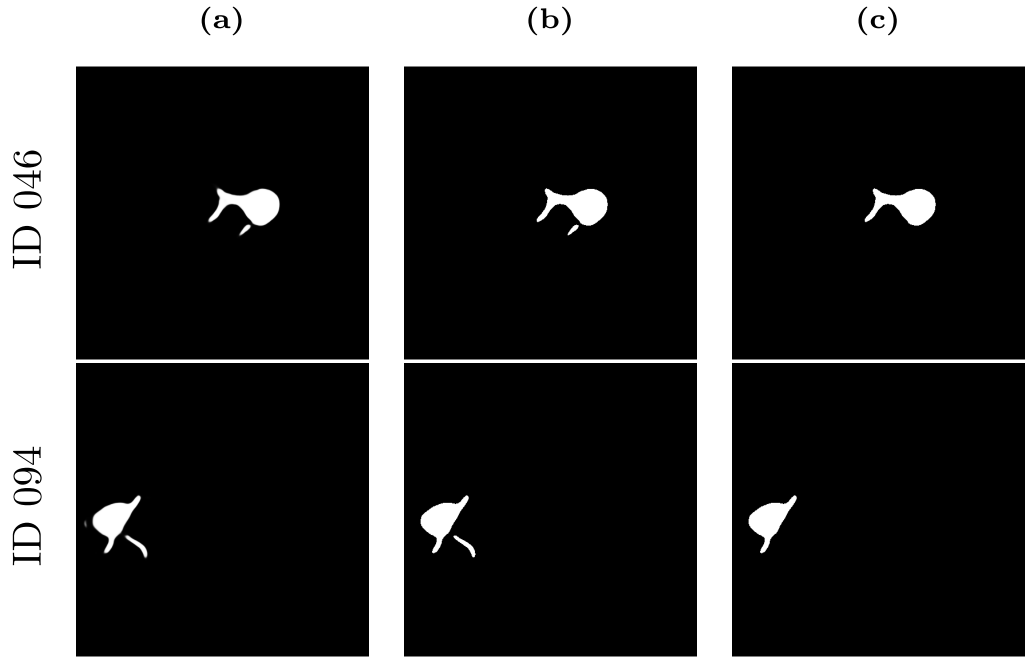

Figure 8. Two examples of using image binarization and clearing to remove vessels that are not connected to MAs. (a) The raw output image of AOSLO-net. **(b)** Output image after binarization. **(c)** Output images after clearing.

3.2 Morphology quantification results for the BNR factor of MAs

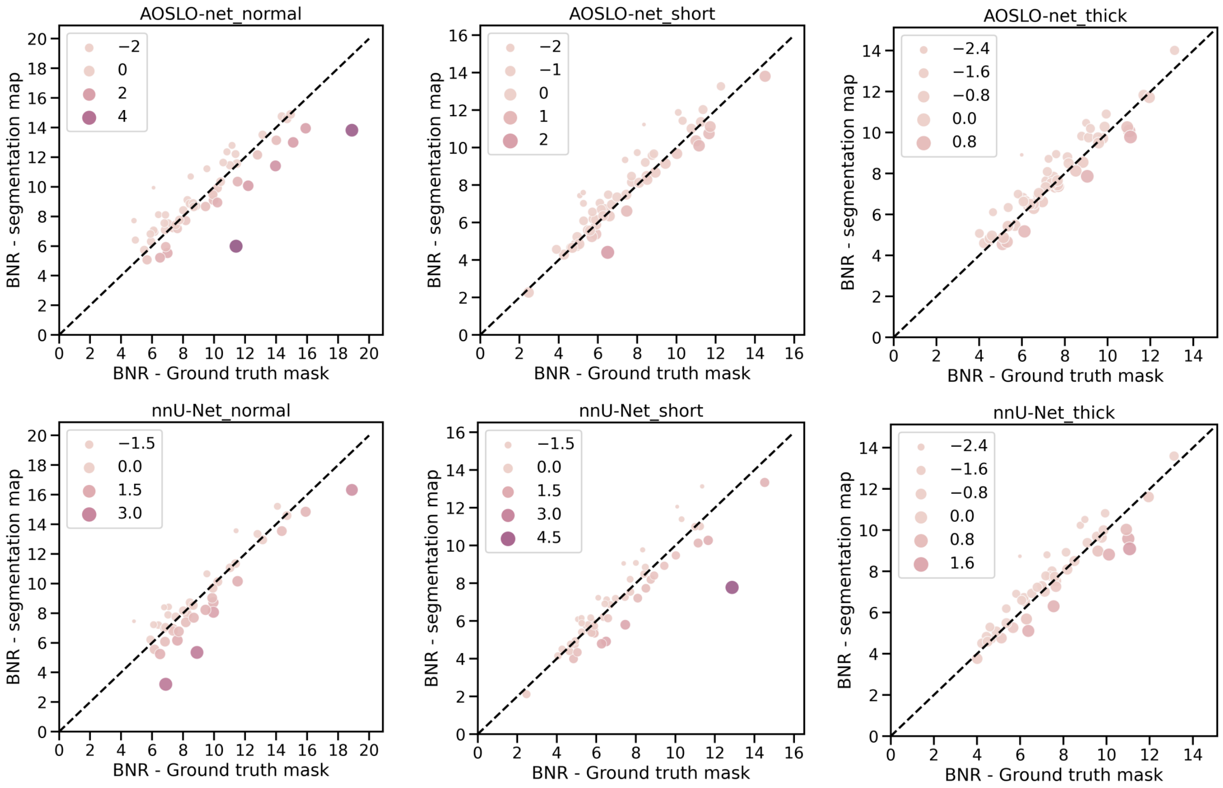

Figure 9. Quantification results of the BNR (body-to-neck ratio) factor for the segmented MAs using AOSLO-net and nnU-Net trained with normal, short and thick masks. First row: Comparisons of BNR quantification results based on AOSLO-net segmentation maps and the corresponding adopted three different types of MA masks (normal, short and thick masks as shown in columns 1–3); **Second row:** Comparisons of BNR quantification results based on nnU-Net segmentation maps and the corresponding adopted MA masks (normal, short and thick masks as shown in columns 1–3). The dot size and color represent the estimated BNR value difference between using the MA segmentation maps and the original ground truth masks. AOSLO-net segmentation maps enable to generate more accurate BNR estimation results (BNR difference values are relatively smaller) in comparison to nnU-Net segmentation maps. Moreover, AOSLO-net trained with thick masks can capture more accurate the MA geometry features (especially for the narrowest calibers). The corresponding BNR estimation results are closer to the BNR values estimated by using the normal masks.

References

[1] Olaf Ronneberger, Philipp Fischer, and Thomas Brox. U-Net: Convolutional networks for biomedical image segmentation. In *International Conference on Medical Image Computing and Computer-assisted Intervention*, pages 234–241. Springer, 2015.

[2] Qiangguo Jin, Zhaopeng Meng, Tuan D Pham, Qi Chen, Leyi Wei, and Ran Su. Dunet: A deformable network for retinal vessel segmentation. *Knowledge-Based Systems*, 178:149–162, 2019.

[3] Pavel Yakubovskiy. Segmentation models pytorch. `https://github.com/qubvel/segmentation_models.pytorch`, 2020. [Online; accessed September-2020].

[4] Jifeng Dai, Haozhi Qi, Yuwen Xiong, Yi Li, Guodong Zhang, Han Hu, and Yichen Wei. Deformable convolutional networks. In *Proceedings of the IEEE International Conference on Computer Vision*, pages 764–773, 2017.

[5] Fabian Isensee, Paul F Jaeger, Simon AA Kohl, Jens Petersen, and Klaus H Maier-Hein. nnU-Net: a self-configuring method for deep learning-based biomedical image segmentation. *Nature Methods*, 18(2):203–211, 2021.

[6] Mingxing Tan and Quoc Le. Efficientnet: Rethinking model scaling for convolutional neural networks. In *International Conference on Machine Learning*, pages 6105–6114. PMLR, 2019.
